# Exploring Swedish veterinarians' awareness of non-accidental-injuries, animal abuse and the Link to domestic violence, and their role in addressing this societal issue

**DOI:** 10.3389/fvets.2024.1439106

**Published:** 2024-12-18

**Authors:** Lisa Oellig, Johan Lindsjö, Helena Röcklinsberg

**Affiliations:** Department of Applied Animal Science and Welfare, Swedish University of Agricultural Sciences, Uppsala, Sweden

**Keywords:** animal abuse, domestic violence, education, non-accidental-injuries, the Link, veterinarian

## Abstract

Research has established a link between animal abuse and domestic violence (the Link), where the perpetrator uses the animal to control the human victim. Veterinarians are exposed to both vulnerable animals and humans, playing a crucial role in detecting and handling these cases. A study using an online survey was conducted in 2019, exploring the awareness of Swedish veterinarians regarding Non-accidental-injuries (NAI*)* in animals, the Link between animal abuse and domestic violence and how to handle such cases. The survey was distributed by the Swedish Veterinary Association to 2,627 licensed veterinarians and was answered by 362 respondents. One of ten had experienced the Link in their clinical work while 63% did not know if they had. The awareness of the Link was generally high (90%), whereas 68% of respondents answered that they did not know or were unsure about the concept of NAI. Almost half of the respondents (44%) felt that they were unsure about how NAI should be diagnosed, 56% did not know how NAI should be documented and a third (34%) did not know which authority should be contacted in case of suspicion of animal abuse. A minority, 17%, knew how to act when there was a reason to suspect domestic violence. The responses showed that support in the clinics is inadequate, with only 10% stating that information material and further education is offered about the Link and 17% knew of action plans or procedures for dealing with a suspected case of animal abuse. Only 16% stated that the Link was part of their education. Improving the ability of veterinarians to act appropriately requires more integrated theoretical and practical training during their university studies. At the clinic, further education, action plans and collaboration with competent authorities and organizations are crucial. It is important that resources, such as information material, guidelines, action plans and educational programs are identified and used to support veterinarians in their work to help vulnerable animals and, by extension, people, in line with the concept of One Welfare.

## 1 Introduction

Reports of Swedish crime statistics by the Swedish National Council for Crime Prevention (Brå) for 2023, show that the total number of reported assault crimes was 61,211, of which 37,909 indicated that they were acquaintances of the offender (1). The statistics also show that there were marked differences between the sexes, with assaults on women by an acquaintance accounting for 81%, while the corresponding proportion for men in the same category was 43%. According to other statistics, women are also more likely to be subjected to more serious violence that leads to medical care and more often than men report that the place where the assault took place was their own home (2). In 2023, ten cases of fatal violence in close relationships were recorded, all of which involved women or young girls (1).

In a survey conducted in 2021, at the request of the Swedish Kennel Club and the insurance company Agria, it was estimated that there were pets in every third Swedish household (3). This means that it is very likely that there may also be pets in homes where domestic violence occurs. According to statistics from Brå, 1,558 cases of animal cruelty were reported in 2023 (4).

The connection between animal abuse and domestic violence (The Link) has been known in research for many years and studies also show that in two out of three cases, women who have been abused in the home have reported that the same applies to a pet and that fear for the animal's safety makes them afraid to leave or seek help (5–8).

See the Link (Swe: Se Sambandet) and Veterinary care for victims of violence (VOOV) (Swe: Veterinär Omtanke Om Våldsutsatta) are two organizations in Sweden that work actively on a non-profit basis to distribute information and knowledge about the Link. VOOV also offers shelter for animals belonging to victims of domestic abuse. Results from studies abroad show that, despite continuous efforts to inform and educate about the Link, there is a large knowledge gap in the identification and handling of cases of domestic violence and animal abuse, especially among animal health personnel (veterinarians, veterinary nurses, etc.), which is a professional group that is very likely to encounter this in their professional role (9–11). We were interested in whether this is also true for veterinarians in Sweden.

The Swedish veterinarians' responsibilities in their professional roles are regulated in the Animal Health and Medical Care Act (SFS 2009:302). As animal health personnel they are obligated to perform their consultation duties in accordance with research and proven experience and to keep accurate documentation of an animal's state of health or care (chapter 2, 1§). Furthermore, it is stated in the Animal Welfare Act (SFS 2018:1192), Chapter 8, 18§, that if animal health personnel finds reason to assume that animals are not being kept or cared for in accordance with this Act, that person shall report this to the control authority, i.e., the County Administrative Board, unless the deficiency is minor and is rectified immediately. This requires an awareness among animal health personnel of the responsibilities and procedures for reporting animal abuse. Animal welfare inspectors employed at the County Administrative Board, execute official animal welfare controls and possible interventions (e.g., removal of an animal from its owner), and report serious breaches to the animal welfare legislation to a prosecutor, however county veterinarians may be involved in the process (12). The animal health personnel may also report cases with obvious animal suffering directly to the Police.

The role of the modern veterinarian mirrors the development of society's perspective on the value and use of domestic animals, but also the development and purpose of the profession itself (13). In general, the view of animals has historically evolved from a more anthropocentric view with human interests in focus, to ascribing pets individual rights and intrinsic value. The primary responsibility of clinical veterinarians relates to their animal patients, but they also have obligations and moral duties to other stakeholders who may have conflicts of interest, such as animal owners, veterinary colleagues or other clinics, and society (14). Thus, the role of the veterinarian is also externally influenced by both economic, professional and political interests, which can risk negatively affecting animal care. This balance highlights the importance of the veterinarian as a key figure in several sections in the One Welfare Framework, which includes the connections between animal and human abuse, and social implications of improved animal and human wellbeing (https://www.onewelfareworld.org/book.html).

The results of a study by Landau (15) examining the role of the veterinarian in identifying and reporting animal abuse, where deans of a number of US and Canadian veterinary schools participated, showed that 97% agreed that veterinarians will encounter cases of animal abuse and 63% that they will also encounter cases associated with domestic violence. Results from similar studies conducted later by Williams et al. (16) in New Zealand and by Sharpe and Wittum (9) showed that 70–80% of veterinarians were aware of the Link and that they felt a responsibility for their animal patients, but that the handling of cases of violence where a human is also a victim is more problematic. Landau's (15) study further showed that 75% of the veterinary school departments included training on the identification and reporting of animal abuse, but only 21% included the same training for cases of human abuse, which can be considered problematic since a large proportion believed that they will also encounter these human cases. In interviews with veterinarians linked to the study, only 7% indicated that they had received education on how to deal with a case of animal abuse and the average time spent in the curricula on animal abuse was estimated to be 76 minutes, and for human abuse only 8 minutes. Since 2017, there is a half-day theoretical training on NAI (Non-accidental-injuries) and the Link in the veterinary program at the Swedish University of Agricultural Sciences (Personal communication J Lindsjö 7 march 2019). The subject is also included in the latest version of the actual course syllabus (17).

This article is based on the results of a master's thesis that aimed to explore Swedish veterinarians' awareness of the Link between animal abuse and domestic violence and of the phenomenon NAI (non-accidental-injuries). Using an online survey, Swedish veterinarians' knowledge of the Link, identification of NAI, important documentation and procedures in suspected cases of animal abuse, and by extension also humans, was mapped. Respondents were also given the opportunity to discuss their own perceived role and responsibility in suspected cases of animal and domestic abuse. Although formulated to describe violence against humans, we find the definition by Isdal (18) is useful also for describing violence against animals, since it explicitly includes the mental dimension, which is prevalent in domestic violence: “Violence is any act which, by frightening, hurting, harming or offending, causes the other to do what they do not want to do or to stop doing what they want to do” (our translation).

## 2 Materials and methods

### 2.1 Web survey

Data were collected from Swedish veterinarians using an online survey. A base of closed questions was chosen to evaluate the proportions of respondents' knowledge and experience on different topics. In order for all respondents to have the opportunity to find a suitable answer option, the answer options “Yes,” “No,” “Don't know”/”Unsure,” “Yes, sometimes” and “Yes, in most cases” were offered. Furthermore, there was an additional question that highlighted respondents' definitions of suffering, with the possibility to fill in one or more (or all) response options.

A pilot survey was sent to 16 respondents from different professional groups to review and test the technical features and wording of the questions. After a slight revision, the final version of the survey was disseminated.

This study combines veterinary medicine, law, ethical reflection and sociological methods and this interdisciplinary approach mirrors the wide range of societal issues raised by NAI and the Link. The responses were analyzed using both quantitative and qualitative methods. The closed questions were analyzed with descriptive analysis to identify frequencies, proportions and patterns and the open questions with personal answers were analyzed to gain a deeper understanding of the respondents' reasoning.

The survey consisted of a total of 19 questions (and a final open comment field) divided into four parts with different themes; About your professional role, About the Link, About injuries and violence and Your workplace—knowledge and routines. In the first part, called “About your professional role”, questions were asked about the number of years worked in the profession, the types of animals worked with and where the respondents had done their veterinary education. These questions were chosen to investigate whether there could be different levels of knowledge, experience and values in the different groups represented.

As the survey was considered to be of interest to the Swedish Veterinary Association (SVF) and its members, we contacted SVF regarding distribution and they were willing to spread the survey. At the end of 2018, SVF had just over 2,600 professional members, 196 students and 639 retirees (Personal communication C Eriksson 5 march 2019) (nb the number of clinically active is unknown to SVF). In 2019, there were 5,378 licensed veterinarians in the country. This means that about 50% of all veterinarians in Sweden are members of SVF. The survey was sent out in spring 2019 to 2,627 members and was available for 3 weeks, during which time one reminder to respond to the survey was sent out.

Respondents were informed in the survey introduction that the survey was anonymous and no personal information would be collected, and that participation was considered as informed consent. Further, given the character of the study we also did not need an ethical approval according to the Swedish Act concerning the ethical review of research involving humans (SFS 2003:460), which states no ethical approval is required if it is not a physical intervention, is not intended to have an impact or to harm the research person mentally or physically and is not a study on biological material.

## 3 Results

### 3.1 Sample group and respondents

The survey was completed by 396 respondents, i.e., a response rate of 15%. Thirty-four respondents were removed from the final results as they failed to complete the majority of the questions, which resulted in a final group of 362 respondents. The majority (89%) were clinical practitioners, but veterinarians from other workplaces also responded, of which a major part had previous clinical experience. The respondents were spread across the country with at least one respondent in each county and the highest represented counties were Stockholm County, Västra Götaland County, Uppsala County and Skåne County, which corresponds well with the distribution of veterinary clinics in the country.

The following information about the professional role of the respondents was used to analyse the responses between different groups at a later stage.

#### 3.1.1 Education

The majority of the respondents, 276 (77%), had completed their veterinary education at the Swedish University of Agricultural Sciences (SLU). Of the remaining 86 respondents, 41 had completed their education at the University of Copenhagen, 44 at different universities within the EU and finally, two respondents studied outside the EU and then supplemented with SLU's supplementary education for veterinarians with a degree from outside the EU/EEA and Switzerland.

#### 3.1.2 Type of veterinarian

The largest proportion of respondents (*n* = 242, 67%) worked with companion animals (dogs, cats, rodents, birds, reptiles, etc.), followed by 76 respondents (21%) who worked with farm animals and horses. The smallest group of respondents answered that they worked with exotic animals (*n*= 2, 1%) and 40 respondents (11%) were not currently clinically active.

#### 3.1.3 Number of active years in the profession

The largest group represented among the respondents were those who had been active for 5–15 years (39%), followed by 25–35 years (19%), 2–5 years (17%), 15–25 years (17%), < 2 years (5%), and the smallest group of > 35 years (3%).

### 3.2 Web survey responses

**Figure 1:**
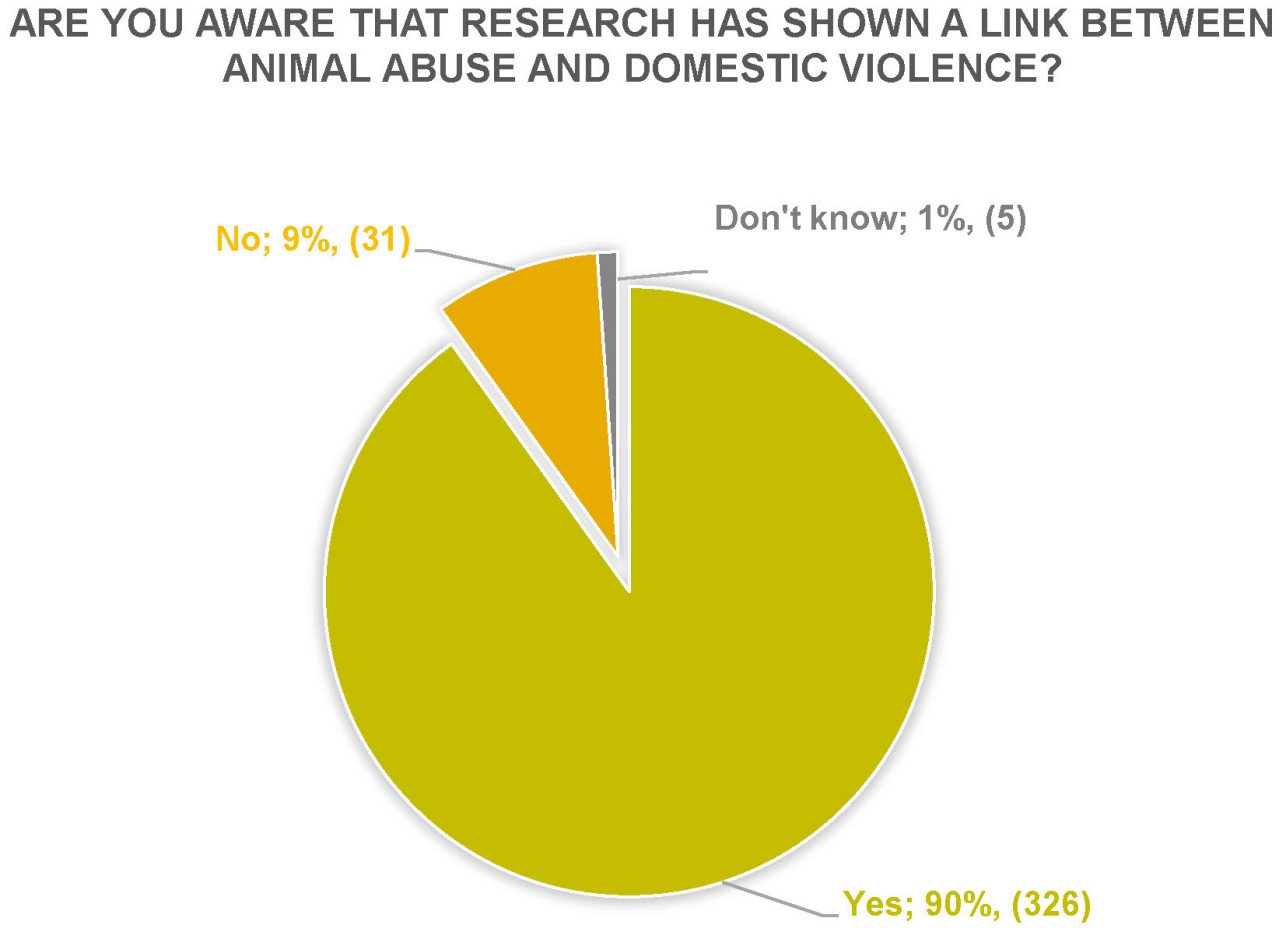
Respondents' answers to the question “Are you aware that research has shown a Link between animal abuse and domestic violence?” *N* = 362.

**Figure 2:**
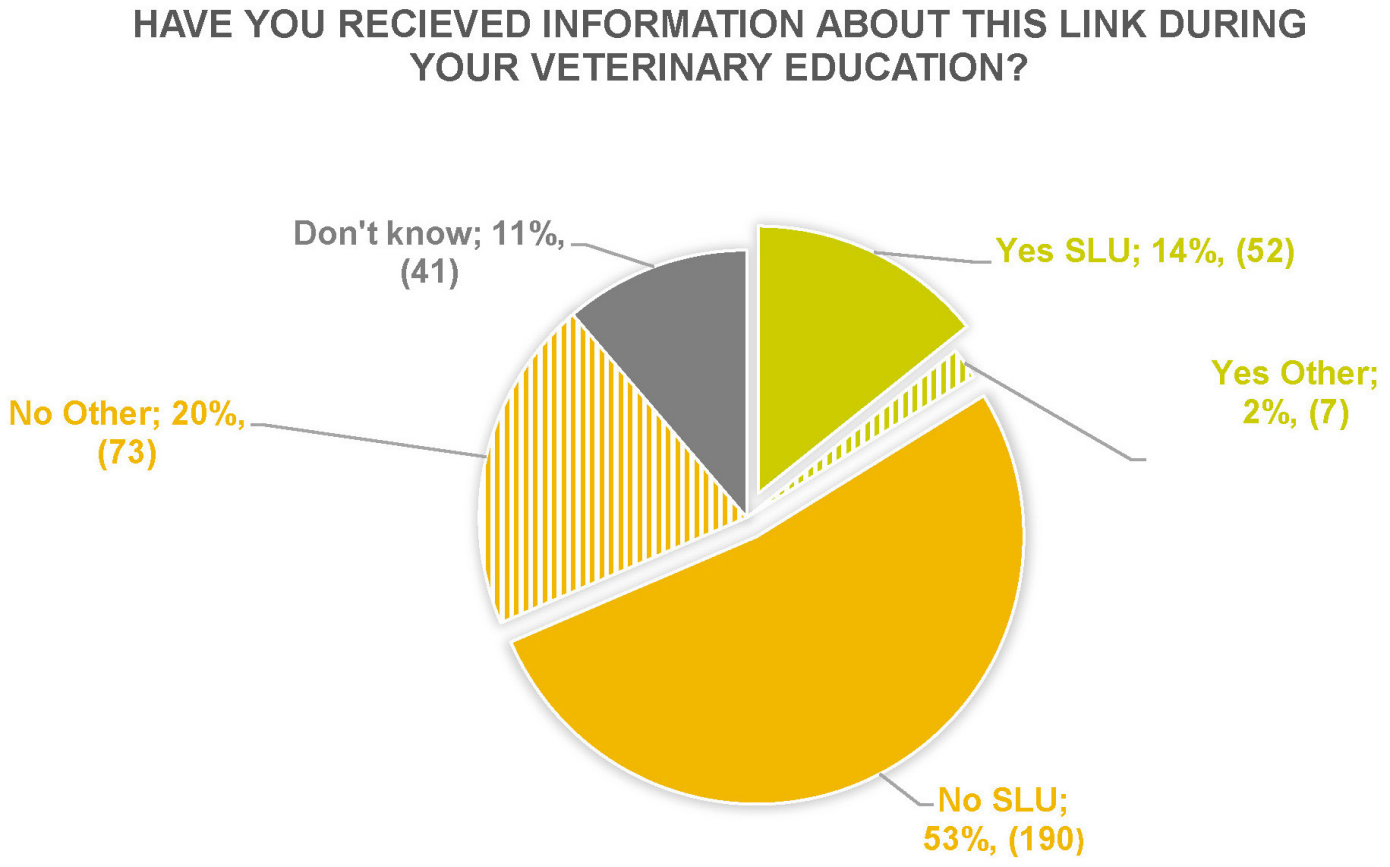
Respondents' answers to the question “Have you received information about this link during your veterinary education?” *N* = 362.

**Figure 3:**
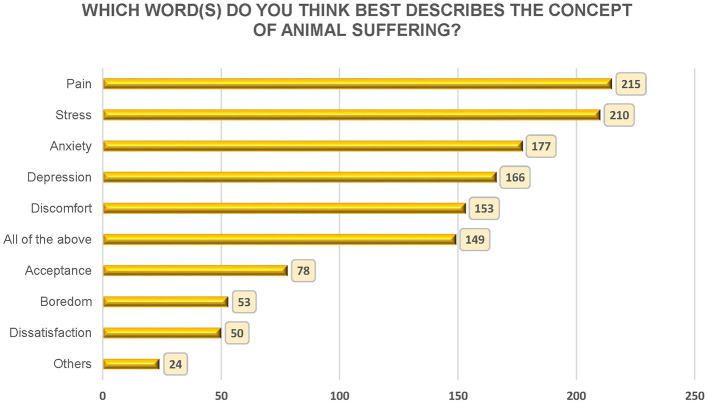
Respondents' answers to the question “Which word(s) do you think best describes the concept of animal suffering?” The number of times each concept was selected is indicated in the bars with the specified number in the boxes. *N* = 362.

**Figure 4:**
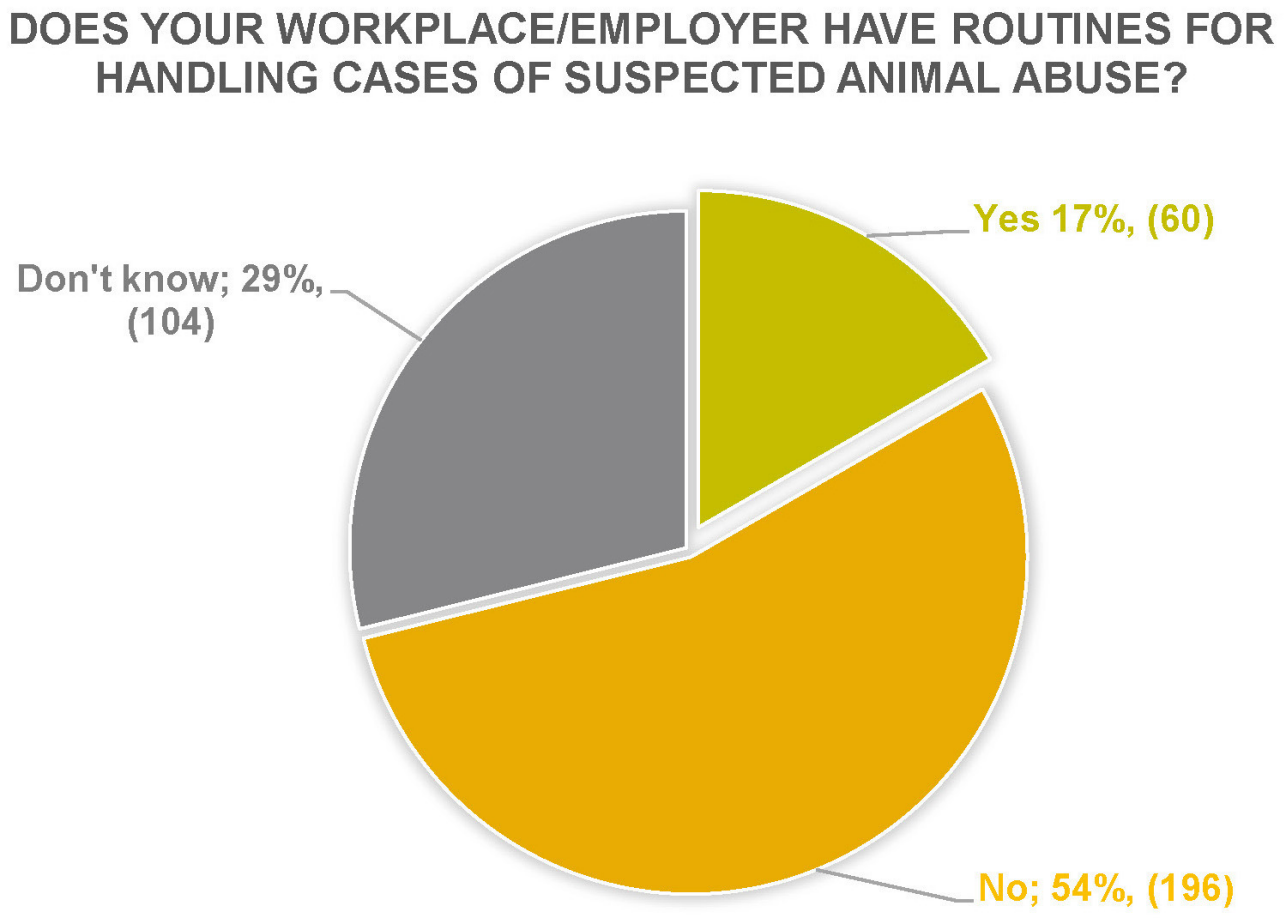
Respondents' answers to the question “Does your workplace/employer have routines for handling cases of suspected animal abuse?” *N* = 360.

The results will be presented under the categories About the Link, About injuries and violence and About your workplace, following the sections in the survey. The quotes used are our own English translations. The results of the survey are presented in Figures 1–4 and Table 1.

**Table 1:** The results of the respondents' answers per question of the entire survey, showed in% and number in brackets, and total number of answering respondents per question (N).

**Question**	**Yes**	**No**	**Don't know/*Unsure***	***N***
**About the Link**				
Are you aware that research has shown a link between animal abuse and domestic violence?	90% (326)	9% (31)	1% (5)	362
Have you received information about this Link during your veterinary education?	14% (52) SLU 2% (7) Other	53% (190) SLU 20% (73) Other	11% (41)	362
Have you experienced this Link in your professional role as a veterinarian?	9% (33)	24% (86)	67% (243)	362
**About injuries and violence**				
Have you previously come across the term NAI (non-accidental-injuries)?	25% (91)	68% (245)	7% (26)	362
Do you consider that you have the skills needed to distinguish between injuries that may be the result of violence and injuries that may be the result of an accident?	17% (61) in most cases 39% (142) sometimes	32% (115)	12% (44)	362
Based on an animal's behavior, do you think you can identify whether it is being mistreated by its owner?	20% (71) in most cases 61% (220) sometimes	13% (48)	6% (22)	361
Have you experienced differences between which animal species are more often exposed to injuries as a result of violence than others?	14% (52)	42% (153)	43% (157)	362
**Your workplace**				
Does your workplace/employer offer education/information about the Link between animal abuse and domestic violence?	10% (37)	71% (257)	18% (66)	360
Does your workplace/employer have routines for handling cases of suspected animal abuse?	17% (60)	55% (196)	29% (104)	360
In the event of a suspected case of animal abuse, do you know which authority to contact?	66% (238)	5% (20)	*29% (104)*	362
In the event of a suspected case of animal abuse, do you know which documentation and evidentiary material that may be important as a basis in any future legal processes?	43% (157)	16% (56)	*41% (149)*	362
Do you consider that you have the skills required to handle a possible perpetrator in a suspected case of animal abuse?	16% (56)	55% (198)	30% (108)	362
In a situation where you suspect that a pet is being subjected to violence (NAI), and you have reason to suspect that a person may also be affected, would you know how to act?	17% (60)	37% (132)	*47% (170)*	362

Numbers in italics show where the response alternative was “Unsure” (instead of “Don't know”).

#### 3.2.1 About the link

##### 3.2.1.1 Are you aware that research has shown a link between animal abuse and domestic violence?

Most of the respondents (90%) indicated that they were aware that there is a Link (Figure 1). One of the respondents who answered Yes, respondent 15, further elaborated in the comments section that they had become aware of the Link through their own past experiences:

“*Own experience of oppressive family fathers whose pets often show nervousness and fear.”*

Of those who answered Yes to the question, reading articles on the subject, discussions in veterinary forums, participation in veterinary congresses and seminars, experienced colleagues, personal contacts and information from VOOV were mentioned as ways in which they had gained this knowledge.

Another respondent who answered Yes commented as follows:

“*I think it's logical. If you use violence, there is a lack of empathy and self-control.”*

- Respondent 94

Three of the respondents who answered No to the question however commented that they were aware of the Link, but not from research, and that it seems logical that there would be a Link.

##### 3.2.1.2 Have you received information about this link during your veterinary education?

The answers showed that the largest proportion (73%) of the respondents had not received training on the Link during their veterinary education and of these, most had studied at SLU (Figure 2). Of the 16% that had answered Yes, almost all had done their training at SLU.

One of the influencing factors mentioned by the respondents who answered No to the question was the time aspect, as they had carried out their education a long time ago when this research was not yet relevant. One of the respondents reasoned about it as follows;

“*That research had not been done, let alone published, when I attended my veterinary education. The link was discovered later, but I have found the information about it in connection with veterinary congresses and through colleagues”*

- Respondent 88

Two respondents who answered No to the question reflected further on the reasons why the information was not covered during their veterinary education;

“*It is possible that it was mentioned during the clinical periods, but overall it was regarded as taboo to talk about and that as a veterinarian you should be on good terms with your pet owners in order not to lose customers”*

- Respondent 141

“*It is general knowledge and does not necessarily belong to my veterinary expertise.”*

- Respondent 213

Respondent 121, who studied their first 3 years abroad, reflected on the possible issues surrounding this as there may be cultural differences in approach and handling of crisis management, pet owner dialogue and critical thinking.

##### 3.2.1.3 Have you experienced this link in your professional role as a veterinarian?

The results showed that 243 (67%) respondents had not experienced the Link, followed by 86 (24%) respondents that had experience and 33 (9%) that were unsure (Table 1). Although the majority of the respondents got this experience from meeting animals at the clinic, a few got this experience as pathologists or during animal welfare inspections as a veterinarian at the County Administrative Board. Respondent 330 described a case where a person called the clinic to inform them that their ex-partner might be bringing their healthy dog to the clinic to be put down as punishment. Respondent 254 talked about their experiences in different professional roles;

“*During my 15 years as a district veterinarian I had cases with terrible conditions for children and then convictions for animal cruelty. Even more examples in the last 6 years as a county veterinarian when handling animal welfare cases where I had to witness strange family relationships.”*

Two other respondents further reflected on the likelihood of people exposed to domestic violence bringing their exposed pets and seeking help:

“*I think that many cases of violence against animals go unreported and there is probably a big amount of hidden statistics. Most of these probably don't come to the vet as it costs money and perpetrators also have a tendency toward financial violence.”*

- Respondent 213

“*I suspect that those who are victims of domestic abuse have much more to think about than even getting to the vet and that we therefore don't see them very often*.

- Respondent 2

Some respondents who answered no, however discussed the problem of working with larger animals that may not be exposed to the same extent or that they were unsure whether they could determine if it was a case of violence against pets or humans.

#### 3.2.2 About injuries and violence

##### 3.2.2.1 Have you previously come across the term NAI (non-accidental-injuries)?

The largest proportion of respondents (68%) answered that they had not encountered the term NAI before (Table 1). Some respondents who answered No and Don't know, commented that they had not encountered this concept in that sense but that they understood the meaning and used other terms to explain the phenomenon. Respondent 14 also highlighted the importance of sticking to a language that lay people can understand and not risk misinterpretation.

Some respondents who answered Yes to the question mentioned that they had encountered the concept in their professional role during a course/conference in Sweden. One of the respondents mentioned a case of an owner suffering from Munchausen by proxy who repeatedly poisoned their dogs and another case where an owner, through an alleged accident, backed over their dog, whereupon the insurance company raised a concern about the sequence of events. One of the respondents who answered Yes to the question reflected on the procedure for suspected cases of NAI;

“*Veterinarians have a responsibility to always defend good animal welfare and human/animal relations, therefore all cases I come across in my professional practice are reported to the county veterinarian for further processing*.

- Respondent 346

Two respondents mentioned that they had come across the term in the media.

##### 3.2.2.2 Do you think that you have the skills needed to distinguish between injuries that may be the result of violence and injuries that may be the result of an accident?

Respondents' answers showed that the largest proportion of respondents considered they had the skill to distinguish between different kinds of causes of the injuries (39%), followed by 32% that did not (Table 1).

A number of respondents commented that in many cases it can be difficult to determine but that it can be investigated by looking at the whole picture: injuries, sequence of events, behavior and abnormalities of the animal and owner, and their interaction. Respondents 2 and 88 commented that one may not be suspicious of one-time occurrences but that repetitive injuries to the same animal may lead to suspicions being raised.

Respondent 1 reflected on the problem of how to identify differences between injuries:

“*Many animals that you meet at a small animal practice, especially cats that go outside, can be exposed to things that we have no idea about; fights, falls from heights, traffic injuries, lacerations, gotten stuck, etc. - where the typical anamnesis is “it came home and was lame, in pain, had wounds, etc.”*

Respondent 345 reflected on the problem of animals behaving differently due to fear of the clinical environment and the difficulty in detecting abusive conditions. Respondent 141 commented that in their basic training there was no education about how to differentiate the injuries clinically and reflected further:

“*As described earlier, I experienced the subject as taboo as the animal owner is a paying customer and in cases where insurance is available, it does not cover damages as a result of self-inflicted violence.”*

One of the respondents who answered No to the question formulated their thoughts as follows:

“*Since we rarely see clear cases of NAI, it is difficult to be sure. It is also in the nature of the veterinary profession and most veterinarians to never be sure of anything nor jump to conclusions.”*

- Respondent 213

Respondent 272 commented that they were very interested in criminology and are therefore usually more suspicious of anomalies and more quickly considers alternative explanations.

##### 3.2.2.3 Based on an animal's behavior, do you think you can identify whether it is being mistreated by its owner?

The largest proportion (61%) of respondents answered that they could sometimes identify mistreatment based on the behavior of the animal (Table 1). Another 20% said they felt they could make the assessment in most cases. According to 29 respondents, with at least one representative from each response option, assessment during clinic visits can be problematic due to lack of time, animals that are uneasy in the clinical setting, very loyal animals that find comfort with abusive owners anyway and differences in species and individuals. Two respondents reasoned about this as follows;

“*I think that animals often change their behavior in the clinical setting compared to their usual environment, which can make this assessment difficult. Weighing in the pet owner's behavior often gives a better picture of the situation.”*

- Respondent 141

”* Many animals are under great stress in clinical settings. In those situations, even an owner who is normally ”nasty“ for the animal, can become its only point of safety. Therefore, it is often difficult to read the animals' reactions correctly in the clinic.”*

- Respondent 79

Respondent 254, who answered No, considered that it is very difficult to see that an animal is unhappy with its owner because animals live in the present and have a forgiving mental disposition. One respondent also commented on the difficulty of detecting abnormal behaviors as there is a risk that it is not the perpetrator who brings the animal to the clinic.

##### 3.2.2.4 Which word(s) do you think best describes the concept of animal suffering?

For the question about animal suffering, eight concepts associated with suffering were presented to the respondents, along with the options All of the above and Other. The question could be answered with any combination of the given alternatives and therefore mainly depicted which concepts were most frequently considered to best explain an animal's suffering. Pain (*n* = 215) and Stress (*n* = 210) were the two most frequently selected concepts (Figure 3).

Four respondents stated that it is difficult to assess suffering in an animal and that in many cases it is a question of degree and duration. Respondent 141 formulated this as follows:

“*Based on our legal system, I would define suffering as a result of the degree of pain and the aspect of time.”*

Respondent 110 further reflected on the difficulty of assessing an animal's suffering:

“*We can only assess animals' emotions based on observable behavior. Stress, anxiety and pain are easy to read based on facial expressions and general behavior. We can never know what the animal really feels, but only assess the animal based on how their emotional life is reflected in their expression and behavior.”*

Some respondents who chose the options Others or All of the above also mentioned other concepts that they felt needed to be added to the list; aggressiveness, fear of beating, stereotypies, anxiety, submissive body posture, malnutrition, frustration, loneliness, fear, thirst, nausea and insufficient opportunities to perform natural behaviors.

Respondent 52 commented that during their education they only learned about compulsive behaviors such as psychological distress in animals:

“*Over time, these will also lead to the development of other veterinary medical conditions. For example cribbing which can lead to thickened neck muscles in horses, as well as licking/biting skin which can lead to dermatitis/bald spots in dogs.”*

A few respondents discussed the issue of psychological suffering and that it should not be underestimated as it can be worse if an animal lives in permanent and prolonged stress or anxiety than if it lives with pain from, for example, osteoarthritis.

Among those who had been working 2–5, 5–15, and 15–25 years, pain was the highest represented concept. In the groups < 2, 25–35, and >35 years, stress was the most represented concept.

##### 3.2.2.5 Have you experienced differences between which animal species are more often exposed to injuries as a result of violence than others?

Only 15% said they had experienced differences in frequency in exposure to injuries between species, whereas there was an equal distribution between those who had not experienced a difference (43%) and those who were unsure (42%) (Table 1). Some respondents commented that it was difficult to know as they only specialize in their species, and therefore have no experience of the prevalence among other species. However, a larger number of respondents commented that they guessed that small animals/pets, especially cats and dogs, are more vulnerable than farm animals. One respondent described their thoughts as follows:

“*Animals that share the home, i.e. are kept within the four walls of the home, are more frequently exposed than those kept outside, for example in stables or free range. Partly, it is a bias that these animals are around more, but it probably depends a lot on the fact that it will go unnoticed inside or that the animals are more tame and you can get close to them. It might also give more emotional leverage to abuse someone's dog than their cattle”*

- Respondent 14

Respondent 110 reasoned along the same lines, believing that there is generally less consideration for the feelings of animals not kept in the house and that farm animals and horses are often treated more harshly than pets, partly because they are not as tame or docile. Furthermore, they felt that it is less socially acceptable to use the same violence against dogs, for example. Respondent 141 mentioned that it might be the case that animals of lower value and status are more likely to be abused and less likely to be taken to the vet. A few of the respondents who had answered No and Don't know, commented that they did not have the experience to make this judgement.

#### 3.2.3 Your workplace

##### 3.2.3.1 Does your workplace/employer offer education/information about the link between animal abuse and domestic violence?

Seventy two percent of respondents answered that their workplace did not offer education or information about the Link, followed by 18% who didn't know and 10% who said it was offered at their workplace (Table 1). Some respondents who answered No commented that there had not been a need or opportunity due to small workplaces or self-employment, that they have never been invited or seen courses/seminars on this topic and that it is not offered if it does not fit in with the workplace competence plan. Some respondents who answered Yes commented that the topic was raised on occasion or that they had the opportunity to attend information evenings, lectures and seminars on the subject. Respondent 13, who answered Yes to the question, reflected on the issue as follows:

“*It is part of everyday life to meet animals in insufficient environments. For example, an insufficient environment for a dog is usually also insufficient for the people who live there.”*

Respondent 329 commented that in many cases their staff are familiar with the situation of the pet owner, in the family and the community and that they had no knowledge of domestic violence. The respondent elaborated further:

“*We sometimes think about this based on the handling of an animal, but it has never been so evident that I think we could proceed with any form of suspicion or report.”*

Two of the respondents commented that their employer had information material about the Link available at the reception and in the restroom and another respondent commented that they had business cards with contact information to women's shelters available in the clinic.

##### 3.2.3.2 Does your workplace/employer have routines for handling cases of suspected animal abuse?

Respondents' answers showed that 54% thought that their employer did not have procedures in place and that a large proportion of respondents were unsure of the situation (29%) (Figure 4). The answers to the question also showed that few workplaces have specific procedures or policy documents to be followed, but that there is good contact with authorities (the County administrative board, animal welfare inspector and county veterinarian, and the police) that can be contacted in case of suspicion. Furthermore, there was also uncertainty about what the workplace offers due to, for example, new recruitment of staff or ignorance of the subject. It was generally considered that guidelines would be a good starting point. Respondent 341 reflected on this as follows:

“*We have no specific routines for this, but in the event of suspicion that an animal is being subjected to suffering of any kind, a report is made to the county veterinarian/police by the veterinarian in charge.”*

Three respondents referred to their obligations according to legislation to report cases where animals are not kept in accordance with the Animal Welfare Act and that they have taken this action in their professional role.

##### 3.2.3.3 In the event of a suspected case of animal abuse, do you know which authority to contact?

The largest proportion of respondents (66%) said they knew which authority to contact, while a third (29%) were unsure and 5% answered No (Table 1). The answers showed that there were differences in how respondents would act in the event of a suspected case of animal abuse, with reporting to the County Administrative Board and/or police, referring to and following workplace policies or contacting the head of the workplace, as being some of the actions they would take. One of the respondents who answered Unsure, knew what applied to the animal in question but reflected further on the Link to domestic violence as follows:

“*Don't know which other authorities you should perhaps contact in view of possible exposed people. Should one even do that or does it fall to the agency I contact with regard to the animal?”*

- Respondent 52

In line with the above, one other respondent commented that in addition to the County Administrative Board and the police, women's shelters should also be contacted.

##### 3.2.3.4 In the event of a suspected case of animal abuse, do you know which documentation and evidentiary material that may be important as a basis in any future legal processes?

The answers showed that the proportion of respondents who felt they had knowledge about necessary documentation (43%) was almost equal to the proportion who said they were unsure (41%), with the remainder answering No (16%) (Table 1). Factors considered by respondents to be of great importance as evidence were accurate record keeping, photographs and description of condition/injuries, documentation of post-mortem findings, laboratory analysis results, assessment of suffering, information about owners and home/environmental conditions. One of the respondents who answered Yes to the question commented as follows:

“*Description of injuries. There is good material from the US for documentation. Pictures and videos of the animal. A detailed journal with a description of the anamnesis and what makes NAI suspected. If possible, a necropsy should be performed, including a well-formulated referral in which any suspicions are stated.”*

- Respondent 118

Four respondents commented that it is also very important that there are witnesses in the form of co-hearing colleagues. Two of the respondents who answered Unsure mentioned the problem of dealing with a possible perpetrator. They commented on the topic as follows:

“*Photographs and a good anamnesis (well-written journal), preferably before revealing that you suspect something so that the animal owner can speak openly and maybe say too much. Examination of the animal where all injuries are carefully and descriptively documented in the medical record. “*

- Respondent 116

“*Yes, but it is not certain that an animal owner will allow further investigation such as blood tests, x-rays or shaving fur to look for visible signs.”*

- Respondent 253

Respondent 120 added that an assessment of the degree and duration of suffering also should be included in the record of the animal.

##### 3.2.3.5 Do you consider that you have the skills required to handle a possible perpetrator in a suspected case of animal abuse?

The largest proportion of respondents (55%) answered that they did not consider themselves to have the knowledge to handle the suspected perpetrator (Table 1). Furthermore, 30% of respondents answered Don't know and 15% answered Yes. The respondents' comments showed that even in cases where knowledge is available, it depends very much on the situation and the atmosphere between the veterinarian and the perpetrator, since threatening situations in the clinic can lead to loss of courage or induce fear. Respondent 245 made the following analogy;

“*Like verbal judo; interpret the situation correctly, do not expose yourself or others to increased risk through provocation. Determined and not backing down when the threats are hailing. Experience and knowledge of psychology and facts regarding what happened, preferably with other people present as buffers so that there are no risky situations.”*

Respondent 247, who answered No, further commented that they would rather have answered both Yes and No and continued as follows:

“*If it is a possible perpetrator who comes with the animal (now I am thinking in clinical practice) I would be very neutral, and then file a report to, if possible, get the animal out of the equation. If it is a possible victim who comes in with the animal, I feel more confident and hope to act in a way that builds trust. For me, the first step has been not to be afraid to raise the issue myself, and I think that VOOV has made it easier to raise the issue because there is also a clear connection to my role as a veterinarian. If I can speak directly to the person I suspect is being abused, I can offer support, refer to VOOV and women's shelters, and if nothing else at least raise the issue to show that someone noticed what is happening. It feels important to not come on too strong with a possible victim, but instead to offer help and solutions within the framework of what is possible for that person at that time.”*

Respondent 248 reasoned about the problem of for example psychopathic personalities, where a perpetrator might have learned to hide possible abusive or manipulative tendencies and be very well-liked and respected in public. In relation to the limited time spent during the visit at the clinic, it can be difficult to detect such tendencies.

##### 3.2.3.6 In a situation where you suspect that an animal is being subjected to violence (NAI), and you have reason to suspect that a person may also be affected, would you know how to act?

Forty seven percent of respondents felt unsure of how to act in case of suspicion that also a person might be exposed, followed by 36% who answered they did not know and 17% who answered Yes (Table 1). Among the respondents who commented further, it was mentioned that they would contact authorities and organizations such as social services, county administrative boards, police, women's shelters, VOOV and churches/parishes. The issue of the victim's safety was also discussed, given concerns about whether the authorities would act in time, the difficulty of separating and speaking undisturbed with the victim, and the fact that in some cases the victim asks the veterinarian not to act for fear of more violence. Respondent 110 who answered No also reasoned about another conflict that may affect the handling of the above cases;

“*It is generally difficult to help people who do not explicitly ask for help, because society thinks personal integrity is more important. There are better procedures to prevent the neglect of animals than of people.”*

Several respondents also mentioned the element of time, contemplating the balance between maintaining a tight consultation schedule and allowing potential victims sufficient time and opportunities to open up and communicate.

##### 3.2.3.7 From your perspective as a professional veterinarian, describe in your own words what obligations you feel you have toward your patients (animals)? Would you feel the same responsibility to act if there were also suspicions of domestic violence?

The ten concepts that were mentioned with the highest frequency among the respondents' free text answers regarding obligations to the animal patient were Suffering (41%), Reporting (34%), Obligation (32%), Responsibility (31%), Suspicion (31%), Act/Intervene (20%), Protect (12%), Animal Advocate (9%), Unnecessary Suffering (9%), and Treatment (6%). The follow-up question on how to act if a human was also a victim of violence was answered by about a quarter of the respondents, of which 67% commented that their main duty is to the animals, but that they would also act if a human was a victim. Further, 24% commented that their duty was equal for the animal and the human and 8% commented that they did not directly consider it their duty to act. The five concepts that appeared with the greatest frequency in their free text answers around this sub-question were Compassion (46%), (more) Knowledge (21%), Moral/ethical responsibility (10%), Women's shelter (7%), and Civil courage (6%). Respondent 95 expressed their thoughts in a way that summarized the overall picture given by the respondents' answers:

“As citizens in our society, we all have an obligation to help and protect each other against violence, threats, injuries, accidents and illness, so-called civil courage. This unwritten law naturally covers the situation of animals as well. The desire for civil courage is governed by upbringing, tradition and legal space. In addition to civil courage, there is legislation for individuals and various professionals that govern what is correct handling in various situations.”

Respondent 256 further reflected on their professional obligation to care for animals in relation to enabling the possibility for the animal to exercise the five freedoms; freedom from hunger and thirst, freedom from discomfort, freedom from pain, injury or disease, freedom to express normal behavior and freedom from fear and stress. Respondent 61 commented that they felt their obligation corresponds well with the student song that was sung during their education, with the name “You are the animals' only hope, you young veterinarian”.

## 4 Discussion

### 4.1 Knowledge of the link

The responses showed that there is generally a high level of awareness of the Link among the respondents, while a majority of the respondents mentioned that they had not received information/training about the Link during their university studies. Irrespective of where the respondents had studied, the majority had not received this information during their education. The half-day training of veterinary students in Sweden started 2017 and it is unlikely that they participated in this study. Based on the number of years they had been working, the answers revealed that the largest number had received information about the Link during their education in the groups that had worked < 2–15 years. This could be explained by the fact that research on the Link has mainly gained momentum during the last 30 years, although there have been people who have discussed the subject much earlier than that (19).

In this study one out of ten veterinarians stated that they had experienced the Link at their clinic, whereas a large proportion of respondents answered that they believed they had not encountered this in their own veterinary clinic/workplace. However, a few of the respondents reasoned that they certainly should have encountered it by now, but most likely have not had the knowledge to identify it. This is also supported by the results of Landau's (15) survey which showed that 97% agreed that veterinarians will encounter cases of animal abuse and furthermore domestic violence (63%), opening up for the suspicion of hidden statistics. The difficulty in identification lies mainly in being able to identify both injuries that may lead to suspicion, but also being able to see suspicious indicators in the behavior of human and animal clients and their interaction, which is also of great importance, according to Arkow and Munro (11) and Munro and Munro (20). Some respondents indicated that they would like to think well of their clients and that they feel the subject is taboo and it is considered negative to report their clients. This phenomenon brings us back to what Sandøe et al. (13) and Rollin (14) have written about the pillars of the veterinarian's role, where client and patient service risks being in conflict with economic and social interests. A study conducted by Kondrup et al. (21), investigating the approaches of veterinary practices toward clients with financial limitations, revealed that, in general, veterinarians act on a basis of relationship-centered care. The respondents in the study demonstrated a willingness to assist particularly vulnerable individuals and animals, especially in cases where strong affection and emotion between the owner and the animal are evident. These findings are in line with the results of this study, where most of the respondents indicated that they would try to act and help an animal and person in need, even in cases where they feel it might not be their professional obligation. Further, this type of reasoning and the sense of responsibility for aiding both humans and animals align with the goals of the One Welfare concept (22). Rather, domestic violence is at its very core. The welfare of an animal is directly linked to the human's wellbeing and possibilities to be a responsible caretaker, and a human whose animal is a victim of domestic violence has impaired wellbeing (that's even the point of harming the animal). One of the current projects at the One Welfare Platform (www.onewelfareworld.org/) focuses on domestic violence.

### 4.2 Assessing injuries and violence

The question about the term NAI showed that the larger proportion of respondents considered that they had not encountered the concept as such and that it was considered difficult to determine differences between NAI and injuries resulting from accidents. It was also seen that there is a great need for education in the identification and diagnosis of NAI both during the veterinary education but also continuing education in the clinics/workplaces. Literature has shown that with careful mapping of the types, appearance, location and age of injuries, it is possible to identify NAI (11, 20). Furthermore, all workplaces should have guidelines in the form of supporting documents to be used as both a procedural checklist and documentation of findings.

The larger proportion of respondents considered themselves to have the competence to assess whether an animal is being mistreated or unhappy with its owner in most cases, unlike the identification of NAI. However, the majority of respondents also commented that identifying deviant behaviors from the normal pattern can be problematic, as many animals can express deviant behaviors due to uncertainty, stress or fear due to the clinical setting. Williams et al. (23) reasoned in a similar way, and also discussed the risk that animal abuse goes unnoticed since it is likely to be “hidden” during veterinary appointments.

The results of the question about which words best described the concept of suffering showed that the most chosen options were pain and stress, which is also depicted in literature showing that mental wellbeing could influence the suffering of an animal to the same extent as physical (24, 25). Suffering is affected by intensity and duration, and factors like previous experiences and expectations (25). In fact, one of the respondents suggested that suffering in the form of, for example, pain from osteoarthritis can be less negative than suffering due to stress. World Animal Health Organization (WOAH) defines suffering as “An unpleasant, undesired state of being that is the outcome of the impact on an animal of a variety of noxious (harmful) stimuli and/or the absence of important positive stimuli. It is the opposite of good welfare.” (26), thus including both mental and physical wellbeing.

Regarding perceived differences between the types of animals that are more likely to suffer injuries as a result of violence, the largest proportion of respondents said that they did not know or that they thought there were no differences, which may be partly explained by the fact that some veterinarians specialize in one or a few types of animals and do not have experience in diagnosing the others. Of the respondents who answered Yes and who commented further, a large proportion mentioned that they believed that small animals and pets were more frequently abused by NAI than other types of animals. This is consistent with research showing that violence against pets is primarily used as a means of exerting power and dominance and requires some type of emotional attachment to the animal, which is more common with pets (27). One respondent also reasoned that there might be economic differences, where a perpetrator might be less likely to victimize animals of higher economic value. However, some respondents reflected on the fact that in many cases farm animals and horses are handled more roughly, often because of their size, which in many cases may not be directly considered as violence. Other studies on domestic violence and animal abuse in rural areas have shown that violence also occurs but on a smaller scale (28, 29). Victims in rural areas may not seek help due to distance and isolation from society, which can lead to many cases of undetected abuse against people and farm animals, which has been considered a major societal problem (28).

### 4.3 The importance of the workplace culture

Respondents' answers to the question about the possibility of education or other information regarding the Link showed that almost three quarters felt that this was not something that was relevant in their current workplace. The reasons for this were considered to be partly related to small workplaces or self-employment, which could mean a lack of resources or time. Another suggested reason was that other areas of competence were regarded more in line with the workplace competence plan and hence prioritized. A high number of respondents considered that they had not encountered a case associated with the Link in their professional role. This may explain the perception that the need for further training does not exist, and consequently, the lack of information material or further education in the clinics.

In terms of procedures for dealing with suspected cases of animal abuse, about half of the respondents said that there were none in their current workplaces, but that they had good relationships with other knowledgeable parties (animal welfare inspectors, county veterinarians and the police) who were considered able to provide information and assistance. That there is a great need for guidelines at the veterinary clinics can also be seen in the literature on the subject, which emphasizes the importance of thorough protocols that clarify the veterinarian's role in relation to and in collaboration with other relevant instances (10). The organization See the Link (Se Sambandet), in collaboration with the The Swedish Association for the Protection of Animals (Swe: Svenska Djurskyddsföreningen) has developed an action plan aimed at veterinary clinics and other animal health personnel to provide guidance in suspected cases associated with the Link (30). The Swedish Veterinary Association has produced guidelines called “Animal welfare in the clinic - Handling of animal welfare cases in small animal clinics” (our translation) and “Handling of animal welfare cases in equine practice” (our translation) which contains detailed checklists, protocols and current legislation that can be used as supporting documentation in cases of animal abuse, as well as information about the Link (31, 32).

In terms of both training or information and workplace practices, a large proportion of respondents felt that they did not know what was available in their own workplace. This may be a result of a lack of information and communication in the workplace, lack of interest in the subject, unwillingness to take on responsibility and uncertainty about their own competences. About one fifth of the respondents said they worked with large farm animals and horses, which may involve other types of risks as these are often treated in the animal's home environment where a potential perpetrator may be present and the veterinarian is often alone. Guidelines and action plans should therefore be adapted to different types of client encounters, taking into account both procedure and available resources.

When asked about the respondents' own knowledge of which authority to contact and what documentation or evidence may be important in suspected cases of animal abuse, the results showed that less than half of the respondents knew what documentation was required and one-third did not know which authority to contact. The fact that one-third of the veterinarians considered themselves not to have knowledge of which authority to contact can be considered alarming given that, according to the Animal Welfare Act (SFS 2018:1192) Chapter 8, §18, they have a duty to notify the control authority when faced with suspected cases of maltreatment. According to the respondents' answers, this could be explained by either an unawareness of the occurrence of animal abuse, or an awareness but with fear of possible risk-taking when reporting, which can lead to negative consequences for the veterinarian or clinic. These arguments are also consistent with conclusions drawn by Arkow (33) in the study on veterinarians' knowledge when suspecting and reporting violence against animals, as well as by Rollin (14) reasoning on ethics and the role of the veterinarian, where they discussed the problem of their own perceived role as a veterinarian and whether their main responsibility is to their clients or animal patients.

When it came to knowledge of proper documentation of abuse, almost half of the respondents said they were unsure what might be important. A large proportion of these, however, gave specific suggestions for factors they thought could be of greater importance, with record keeping and social skills/basic knowledge of psychology being highlighted in particular.

In terms of competence to respond appropriately to a possible perpetrator in a suspected case of animal abuse, just under a fifth of respondents said they considered themselves competent in such a situation, whereas half of the respondents said they were unsure how to act in a case where it is suspected that both an animal and a human may be victims of abuse. These results can perhaps be explained by the fact that reporting domestic violence is not perceived as a core responsibility for veterinarians working in Sweden. It is important though, from both a safety point of view and a social competence perspective. Regarding safety, the veterinarian may end up in a relatively vulnerable situation when forced to meet a perpetrator in a clinical context. Regarding social competence, high skills and training can contribute to gaining as much information as possible, in order to find ways to save the victims (human and animal) from further threats and abuse. This is consistent with findings from a workshop conducted with veterinary students in New Zealand in 2020 that addressed this issue, where 48% of participants indicated that they did not feel safe dealing with a case of suspected animal and human abuse, with words such as fear, revenge, conflict and provocation being some of the words used to describe the problem (34). The reference to lack of knowledge is also consistent with statements made by veterinary respondents in Sharpe and Wittum's (9) study, where 84% cited lack of training to prepare for such situations as a reason for not acting, despite the feeling that it is a professional obligation to help abused animals and in extension victims of domestic abuse.

Further, our respondents' discussion focused on the extent to which it is possible to help a victim without jeopardizing their safety, and also that it can be difficult to help people who do not explicitly ask for help, where personal integrity is an important factor. One respondent mentioned that their own experience of domestic violence has led to an awareness of the Link and the warning signs that exist, and that they are more observant and can more easily get a victim to confide in them.

A study by Arkow (33) examined what veterinarians need to know in order to correctly identify and respond to suspected cases of animal and human violence. The study mentioned a number of steps that have been used in humans to deal with, for example, violence against children, the elderly and intimate partners. Arkow believes that these steps in the form of clinical findings, behavioral science and knowledge of warning signs could be taught and applied to animal abuse as well. Implementing new routines, guidelines and working methods in a workplace places great demands on an awareness of the subject and a willingness to cooperate, and the success of the implementation is also affected by the prevailing organizational culture (35, 36). Successful implementation also includes continuous competence development regarding NAI and the Link in the clinic/workplaces and keeping up with the latest research in the area.

### 4.4 Attention and care—Core elements in the ethical responsibility of the veterinarian

According to the respondents' free text answers to the question about their own perceived responsibility in their role as a veterinarian, the most frequently mentioned concepts were: suffering, reporting, obligation, responsibility, suspicion, act, protection, animal advocacy, unnecessary suffering and treatment. These concepts paint a picture of their moral standpoints and considerations in relation to their own role as veterinarians and further in their own perceived role as fellow human beings and in society.

Most probably without any purpose to do so, the respondents thereby use general concepts that mirror core lines in animal ethics theories or perspectives without considering which one, or any specific one. Moreover, the way they formulated their thoughts, links well to a key concept of an animal ethics of care approach, attention, which emphasizes the importance of seeing the individual and their needs and involves caring for weaker or vulnerable individuals (37). Other concepts mentioned by the respondents also fit well into reasoning in line with the animal ethics of care approach, where relieving, paying attention, caring, curing, empathy and trust can be linked to a desire for deeper understanding of the situation of both animals and humans (One Welfare), often combined with a desire to help the victims away from a violent home situation. According to this type of approach, humans have a responsibility to act on two levels: both for the victims, and for improvement of societal and political structures. This corresponds well with the view of respondents who stressed the obligation to report suspected violence to the authorities, as a means to increase societal awareness and contribute to statistical mapping.

Ethical duty, rights, five freedoms and animal welfare are some other concepts that were mentioned, which can instead be linked to reasoning more in line with the *Animal Rights Ethics* perspective proposed by Cochrane (38). He argues that animals are considered to have the right not to be physically or mentally harmed, or to be killed, but no right to full liberation. That is, respecting animal rights does not imply that animals have a right not to be used at all, they may well be kept for and by humans, but this involves a corresponding duty to treat them well. Furthermore, some respondents instead leaned somewhat toward the preference for preserving customer relationships and economic status rather than taking on an obligation to report suspected animal abuse. This reasoning reveals some contractarian strains, but their rationale of keeping a customer satisfied, as well as the overall picture of the views presented by these respondents rather indicate ways of thinking that are more linked to *Utilitarian Ethics* by focusing on the pursuit of the greater good even at the cost of an individual animal or human (39).

The most frequently used concepts in response to the follow-up question on whether respondents felt the same obligations to act in situations of violence against humans were compassion, more knowledge, moral responsibility, women's shelter and civil courage. The use of these concepts show that many respondents reasoned along the line of *Virtue Ethics* i.e., stressing their individual moral responsibility to act, although they do not have any professional obligation to report cases of abuse involving humans.

Rollin (14) emphasizes that the important role of the veterinarian in detecting pet and human abuse means that training in ethical analysis and balancing ethical aspects is and should be an important part of their education, in order to get the ability to properly weigh and compare the interests of the animal, the owner, the economy and applicable legislation. Skills in ethical reasoning is also a “Day One Competence” according to the Directive 2013/55/EU (Annex 1) on veterinary education, and could hence be expected to be part not only of the current curricula, but also of factual capacities among veterinarians educated thereafter.

### 4.5 Important work yet to be done—Education, awareness, and collaboration

The respondents' answers to the survey indicate that there are three main areas where development could help veterinarians in their professional role to better identify and deal with cases of animal and human abuse. Firstly, veterinary students should receive a good basic education in these subjects. Subsequently it is important to promote further competence development, i.e., education and training, and implementation of guidelines in the veterinary clinics and lastly to strengthen the cooperation between the veterinary clinics and other stakeholders that are involved.

The implementation of the half-day training in Swedish veterinary education in 2017 marks a positive initial step but can still only be seen as an introduction to the subject and above all focusing on theoretical knowledge of the subject. Development of practical sessions in pathology and clinical parts of the education are very important, especially in the diagnosis of NAI, but also integrating the Link. Furthermore, integration between theoretical and practical sessions and between different programs (veterinary, veterinary nursing, ethology and animal welfare, agronomy) using fictional cases connected to their future professional roles could allow for a more realistic context which prepares for future interaction and collaboration. In addition, education in basic psychology and how to act and communicate with animal owners or possible perpetrators is also vital to minimize risks for the veterinarian. A study has shown that there are some collaborations between domestic violence shelters and veterinary schools in the US, where the curriculum includes clinical rotations, lectures and extra-curricular opportunities associated with the Link, resulting in that most of the students after their education felt that their knowledge of the subject had increased (40).

The high proportion of respondents in this study who stated that the workplace does not offer information/education and lacks procedures and guidelines shows that the veterinary clinics are not prepared and veterinarians not properly prepared for identifying cases of violence against animals and/or humans and furthermore do not receive the support that may be needed. It is important to consider possible reforms of the organizational culture and that the subject should not be taboo. Information material should be produced or retrieved from other available sources, both for internal and external use, in the form of action plans for employees and information material for customers (victims) that can be posted in restrooms or at the reception desk. In Sweden, action plans, guidelines and other educational material can be found at the Swedish Veterinary Association, See the Link (Se Sambandet), VOOV and Västmanland County Administrative Board, among others. Paterson et al. (41) conducted an exploratory study of Australian veterinarians' knowledge of how to recognize, respond and refer cases of domestic violence linked to animal abuse, where the results showed that veterinarians who had undergone their specific training felt that they could deal with these cases with better confidence. Similar to this study, their results further show that veterinarians are a very important resource in the identification of animal and human abuse and that factors such as further education and collaboration within and between clinics and other instances strengthen veterinarians in these roles.

In Sweden there are a number of instances that veterinarians and other animal health personnel can contact in case of suspicion of domestic abuse. For suspected cases of children abuse, social services should be contacted. There are a number of organizations for abused women and girls i.e., Womens shelters and the national womens helpline (Swe: Kvinnofridslinjen), and for men there is a similar support helpline. VOOV is to date the only organization in Sweden that offers the possibility to temporarily rehome animals belonging to people exposed to domestic violence.

In 2016, the County Administrative Board of Västmanland set up the project “Animals and Violence” funded by a government grant. It was a collaboration group with representatives from the Police, social services, VOOV, Evidensia Specialist Hospital Strömsholm, women's shelters, the Center against Domestic Violence (CMV) and Mälardalens Women's Refuge (42). The group spread information about the Link and developed a collaboration plan, education material, procedures and network possibilities for relevant instances. The project led to an increased awareness of violence against animals and the Link, both among various authorities and animal health personnel, which in turn led to better collaboration and a higher quality of reports and thus a better basis for better decisions and interventions. Although the evaluation of the project with the so-called “Västmanland model” showed positive results, the financial governmental support was time restricted and the collaboration was to a large extent discontinued and hence the aim of a national standardization has not been achieved.

Mobilization of different authorities and information about who is responsible for what in an ongoing case of animal or human abuse, could lead to veterinarians being more confident about their roles, as well as knowing where support and help is available in case of problems (10). The fact that the “Västmanland model” was so successful shows both a great interest and commitment to making a difference for the good of animals and humans, but also that in many organizations today, external support is required to start up such a process and that this should be a priority that is driven forward also in politics at both local, regional and national levels.

One limitation of this study was that the overall response rate of the survey was quite low, and therefore, the results can only be seen as an indication of the situation among Swedish veterinarians. However, since the results still showed a large overall lack of knowledge regarding identification of NAI and available routines, as well as limited information materials in the workplaces, it could be argued that the proportion that did not answer might be less interested in or aware about the topic and hence a higher response rate might have shown even lower overall awareness and knowledge.

## 5 Conclusion

The respondents' answers show that there is generally a high overall awareness of the Link between animal abuse and domestic violence, but that there is a lack of knowledge about how to handle such a case due to uncertainty about identifying NAI and human abuse, dealing with victims and perpetrators and a concern about negative consequences for the veterinarian or the clinic when reporting clients to the authorities. Respondents' knowledge of their obligations to report animal abuse, which authority to contact and their reasoning about reporting human abuse shows that despite lack of knowledge, most see a moral obligation to follow the law, showing attention to the individual animal based on empathy with their fellow humans and animals.

There is a significant need for education and subsequent skill development, both theoretical and practical, both in the Swedish veterinary education and in veterinary clinics. The development and use of guidelines, action plans and support material regarding animal abuse, NAI and the Link should be prioritized in veterinary clinics and collaboration between the various stakeholders involved in these cases should be organized to clarify roles and responsibilities.

In line with the One Welfare approach, we hope this study contributes to highlighting the need of skills and competence to make a proper diagnosis for disclosing cases of NAI and domestic violence, and the importance that the clinic is prepared and equipped to assist vulnerable animals and thereby humans who are victims of domestic violence.

## Data Availability

The raw data supporting the conclusions of this article will be made available by the authors, without undue reservation.
