# Supplementary material for: Exploring Swedish veterinarians' awareness of non-accidental-injuries, animal abuse and the Link to domestic violence, and their role in addressing this societal issue
Source: Front Vet Sci. 2024 Dec 18;11:1439106. doi: 10.3389/fvets.2024.1439106 (PMC11688811; doi:10.3389/fvets.2024.1439106)
Supplement: Supplementary file 1 [file Data_Sheet_1.pdf]

Oellig. Lindsjö, Röcklinsberg

Attachment to manuscript submitted 2024 05 27*: Exploring Swedish veterinarians' awareness of Non-accidental injuries, animal abuse and the Link to domestic violence, and their role in addressing this societal issue*" (No 1439106): **Survey questions translated from Swedish to English**

See the Link!

Hello and welcome! This survey is produced as part of a masters project and can be seen as part of the efforts to increase society's awareness of the Link between animal abuse and domestic violence. This time it is the veterinarians' turn!

Research has shown that there is a link between animal abuse and violence against women and children in close relationships. Veterinarians can play a significant role in detecting such violence, as in their professional roles they meet animal owners and have the opportunity to detect both animals and people exposed to violence, at an early stage. The purpose of this work is thus to investigate what knowledge Sweden's veterinarians have on the subject, as well as what procedures are in place to handle such cases.

The survey is completely anonymized and consists of a total of 20 questions divided into 3 parts with different themes. Each part has about 3-6 questions, most of which are checkbox questions where one option is selected. There are also a few multiple choice and open comment field questions, as well as the opportunity to write your own comments under each question. Each comment field has an icon that gives examples of what might be important for the specific question.

I look forward to receiving your responses and am very grateful for you taking the time to complete this survey!

About your workplace

Question 1a. At which clinic/practice are you employed?

____

Question 1b. In which county is this clinic/practice located?

Dropdown menu with all the counties in Sweden

Question 2. At which University did you receive your veterinary education?

SLU ☐

Other [ ]

Question 3. Which species do you work with?

Choose the option that you work with the most.

Farm animals (horse, cattle, pigs, sheep, goats etc.) ☐
Pets (dog, cat, rodents, birds, reptiles etc.) ☐
Exotic animals (zoo animals, wild animals etc.) ☐

Other ☐

Question 4. How long have you been active in your profession?

- < 2 years ☐
- 2-5 years ☐
- 5-15 years ☐
- 15-25 years ☐
- 25-35 years ☐
- > 35 years ☐

About the Link

This section includes questions about your knowledge of the Link between animal abuse and domestic violence. Please select one answer option per question and feel free to elaborate in the following comment fields. Below each comment field are examples of what type of information that is important for the specific question.

Question 5. Are you aware that research has shown a Link between animal abuse and domestic violence?

- Yes ☐
- No ☐
- Don't know ☐

Comment

___

**For example, you can describe how and where you obtained this information.*

Question 6. Have you received information about this Link during your veterinary education?

- Yes ☐
- No ☐
- Don't know ☐

Comment

___

**For example, you can describe the context of your education and how you experienced it.*

Question 7. Have you experienced this Link in your professional role as a veterinarian?

- Yes ☐
- No ☐
- Don't know ☐

Comment

___

**For example, you can describe these experiences in more detail here.*

Attachment to manuscript submitted 2024 05 27*: Exploring Swedish veterinarians' awareness of Non-accidental injuries, animal abuse and the Link to domestic violence, and their role in addressing this societal issue*" (No 1439106): **Survey questions translated from Swedish to English**

About injuries and violence

This section includes questions about your knowledge of injuries and violence against animals. Feel free to answer both the checkboxes and the comment boxes. Question 10 can be answered with several options. Below each comment field you will find examples of what type of information that is important for the specific question.

Question 8. Have you previously come across the term NAI (Non-Accidental-Injuries)?

- Yes ☐
- No ☐
- Don't know ☐

Comment

___

**For example, you can describe your knowledge of the concept and the context in which you encountered it.*

Question 9. Do you think that you have the skills needed to distinguish between injuries that may be the result of violence and injuries that may be the result of an accident?

- Yes, in most cases ☐
- Yes, sometimes ☐
- No ☐
- Don't know ☐

Comment

___

**For example, you can describe how you distinguish between these types of injuries.*

Question 10. Based on an animal's behavior, do you think you can identify whether it is being mistreated by its owner?

- Yes, in most cases ☐
- Yes, sometimes ☐
- Sometimes ☐
- No ☐
- Don't know ☐

Comment

___

**For example, you can describe which these behaviors might be and how you identify them.*

Question 11. Which word(s) do you think best describes the concept of animal suffering?
(Choose one or more answer options)
** If you check this option, write the words you want to add in the comment field.*

- Pain ☐
- Discomfort ☐

Dissatisfaction ☐
Depression ☐
Boredom ☐
Acceptance ☐
Stress ☐
Anxiety ☐
All of the above ☐
Others* ☐

Kommentar

___

**Here you can elaborate on the options you have chosen and describe the others you want to add.*

Question 12. Have you experienced differences between which animal species are more often exposed to injuries as a result of violence than others?

Yes ☐
No ☐
Don’t know ☐

Comment

___

**For example, you can describe what differences you have seen and why you think this might be the case.*

Your workplace

This final section consists of questions about routines in your workplace and your knowledge of how to respond to suspected cases of animal abuse. Feel free to answer both the checkboxes and the comment fields. Question 19 is an open question that is answered in your own words in the comment field. The last part (20) is open for your other comments and reflections on the topic.

Question 13. Does your workplace/employer offer education/information about the Link between animal abuse and domestic violence?

Yes ☐
No ☐
Don’t know ☐

Comment

___

**Here you can describe in what way this is offered.*

Question 14. Does your workplace/employer have routines for handling cases of suspected animal abuse? *(If yes, please describe these in the comments field)*

Yes ☐
No ☐

Don't know ☐

Comment

____

**Here you can describe which routines are in place, how you think they work and whether any should be added.*

Question 15. In the event of a suspected case of animal abuse, do you know which authority to contact?

Yes ☐
No ☐
Unsure ☐

Comment

____

**For example, you can describe which authority you would contact and in which situation you contact them.*

Question 16. In the event of a suspected case of animal abuse, do you know which documentation and evidentiary material that may be important as a basis in any future legal proceedings?

Yes ☐
No ☐
Unsure ☐

Comment

____

**For example, you can describe what type of documentation/evidence you think is important and why.*

Question 17. Do you consider that you have the skills required to handle a possible perpetrator, in a suspected case of animal abuse?

Yes ☐
No ☐
Don't know ☐

Comment

____

**For example, you can describe how you would proceed and what kind of skills might be needed in such cases.*

Question 18. In a situation where you suspect that an animal is being subjected to violence (NAI), and you have reason to suspect that a person may also be affected, would you know how to act?

Yes ☐
No ☐
Unsure ☐

Comment

___

**For example, you can describe here how you would proceed in such a situation.*

Question 19. As a professional veterinarian, describe in your own words what obligations you consider you have towards your patients (the animals)?
For example, you could describe how you respond to the suffering of an animal and what responsibilities you feel you have in dealing with cases of violence against animals. Do you feel the same responsibility to act if there are also suspicions of violence against humans?

___

___

20. Other comments and thoughts on the subject.

___

___

___

Please double-check before submitting to make sure all questions are answered. Your participation is important in raising veterinary and societal awareness of the Link.
Thank you for your time and commitment!
